# Proteome Analysis of Walnut Bacterial Blight Disease

**DOI:** 10.3390/ijms21207453

**Published:** 2020-10-09

**Authors:** Cíntia H. D. Sagawa, Renata de A. B. Assis, Paulo A. Zaini, Phillip A. Wilmarth, Brett S. Phinney, Leandro M. Moreira, Abhaya M. Dandekar

**Affiliations:** 1 Department of Plant Sciences, University of California, Davis, CA 95616, USA; chdsagawa@ucdavis.edu (C.H.D.S.); redab@ucdavis.edu (R.d.A.B.A.); pazaini@ucdavis.edu (P.A.Z.); 2 Departamento de Ciências Biológicas, Instituto de Ciências Exatas e Biológicas, Núcleo de Pesquisas em Ciências Biológicas, Universidade Federal de Ouro Preto, Ouro Preto 35400-000, Brazil; lmmorei@gmail.com; 3 Proteomics Shared Resource, Oregon Health and Science University, Portland, OR 97239, USA; wilmarth@ohsu.edu; 4 Proteomics Core Facility, University of California, Davis, CA 95616, USA; bsphinney@ucdavis.edu

**Keywords:** *Xanthomonas*, walnut blight, fruit, proteomics, disease susceptibility, adaptation, LC-MS/MS, virulence

## Abstract

The interaction between the plant host, walnut (*Juglans regia*; Jr), and a deadly pathogen (*Xanthomonas arboricola* pv. *juglandis* 417; Xaj) can lead to walnut bacterial blight (WB), which depletes walnut productivity by degrading the nut quality. Here, we dissect this pathosystem using tandem mass tag quantitative proteomics. Walnut hull tissues inoculated with Xaj were compared to mock-inoculated tissues, and 3972 proteins were identified, of which 3296 are from Jr and 676 from Xaj. Proteins with differential abundance include oxidoreductases, proteases, and enzymes involved in energy metabolism and amino acid interconversion pathways. Defense responses and plant hormone biosynthesis were also increased. Xaj proteins detected in infected tissues demonstrate its ability to adapt to the host microenvironment, limiting iron availability, coping with copper toxicity, and maintaining energy and intermediary metabolism. Secreted proteases and extracellular secretion apparatus such as type IV pilus for twitching motility and type III secretion effectors indicate putative factors recognized by the host. Taken together, these results suggest intense degradation processes, oxidative stress, and general arrest of the biosynthetic metabolism in infected nuts. Our results provide insights into molecular mechanisms and highlight potential molecular tools for early detection and disease control strategies.

## 1. Introduction

Walnut (*Juglans regia*) is an economically important specialty crop, providing edible nuts, high-quality wood, and medicinal uses, with a distribution ranging from tropical to temperate zones in Asia, Europe, and the Americas [1,2,3]. It is among the most-produced nuts in the world, together with almonds, hazelnuts, pistachios, and cashews [4]. Walnuts are an excellent source of plant-based protein, fiber, magnesium, polyphenols, and α-linolenic acid, a plant-derived omega-3 fatty acid [5,6]. The regular consumption of walnuts has been linked to positive health outcomes, including improved cognitive function, reduced cancer, diabetes, and weight control, as well as heart, gut, and reproductive health [7,8,9,10,11,12,13,14], as part of the “Mediterranean diet” [15,16]. The increasing demand for walnuts has supported the robust growth in global nut production [4], reaffirming the increasing importance of the walnut industry. 

Walnut bacterial blight (WB) is a bacterial disease that reduces the productivity of walnut orchards in California and worldwide [17]. This disease decreases the quality, especially in wet years and in early-leafing varieties. Walnut blight is caused by *Xanthomonas arboricola* pv. *juglandis* (Xaj), a gamma-proteobacterium that attacks various tissues of English (Persian) walnuts, including catkins, female flowers, fruit, green shoots, buds, and leaves [18]. The primary cause of economic loss is disease development in the fruits after flowering. One of the challenges of controlling the bacterial spread is the extended period of host susceptibility. The pathogen can survive in infected trees in twig lesions, buds, and diseased fruits year after year [19]. The biology of the host-pathogen interactions and the underlying molecular mechanisms that mediate the virulence, increased susceptibility, and/or resistance of the host plant have not been characterized. This lack of knowledge leads to broad-spectrum and ineffective measures of disease control in the field. Current control strategies spray orchards with copper-based biocides in combination with EBDC (ethylene bis-dithiocarbamate) fungicides and time the applications with disease-prediction modeling, such as XanthoCast [20,21]. The inability to predict the disease at asymptomatic stages remains a major challenge for walnut growers. However, an increasing resistance to copper-based biocides in natural Xaj populations and an increasing dependence on EDBC fungicides for control trigger both immediate and long-term concerns regarding the viability of the continued registration of these compounds, due to their accumulation in the environment and toxicity [22].

Copper-based biocides are effective in many pathosystems involving *Xanthomonas* spp., although some Xaj isolates are especially resistant [23,24]. Some pathosystems such as the bacterial blight of rice, bacterial spot, and black rot diseases have been studied in molecular detail, revealing specific plant-microbe protein interactions [25,26,27,28,29]. These include, for example, production by the host of pathogenesis-related (PR) proteins triggered by detection of pathogen- or damage-associated molecular patterns (PAMPs and DAMPs, respectively) [30,31]. Another layer of responses involves effector-triggered immunity (ETI) upon the detection of type III effectors secreted into plant cells by the bacterium, capable of altering the host gene expression and disease progression [32]. Similar strategies are also employed by *Xanthomonas* pathogens in stone and fruit trees, exemplified by citrus bacterial canker disease caused by *Xanthomonas citri* [33]. Despite these similarities, there are also significant differences such as the adoption of transcription activator-like (TAL) effectors to alter host responses by some, but not all, *Xanthomonas* species [34]. Hence, the need to study each pathosystem in molecular detail, so common and disease-specific themes can be identified and characterized more precisely. In WB, a distinctive symptom on fruits is the intensely dark pigmentation of the hull tissues that progresses to the edible parts, until the kernel is damaged [17]. Polyphenol oxidases (PPOs), which are copper-containing enzymes, have been implicated in the synthesis of these pigments, which include tannins and melanins [35,36,37], and have attracted further interest in their role in plant health [38]. 

Defining the molecular markers that can assist molecular breeding or gene-editing approaches is a necessary step to accelerate breeding efforts for tree crops. WB is among the few pathosystems in which complete genome sequences of both the host and pathogen are available as a valuable reference [39,40], contributing to the molecular phenotyping of the disease process. To provide an in-depth assessment of the molecular responses and interactions between the host and pathogen leading to WB, we performed a comparative proteomic analysis of symptomatic vs. healthy fruit tissues. By combining state-of-the-art LC-MS/MS and data analysis methods for peptide identification, the major molecular functions and biological processes affected by WB were determined, as were the individual proteins and protein families with differential abundance. These molecular networks deepen our understanding of the role of specific proteins and protein classes in plant-pathogen interactions and reveal details of the disease mechanisms behind WB. This can potentially lead to more targeted and sustainable therapies to protect walnut production against diseases like WB.

## 2. Results

**Figure 1:**
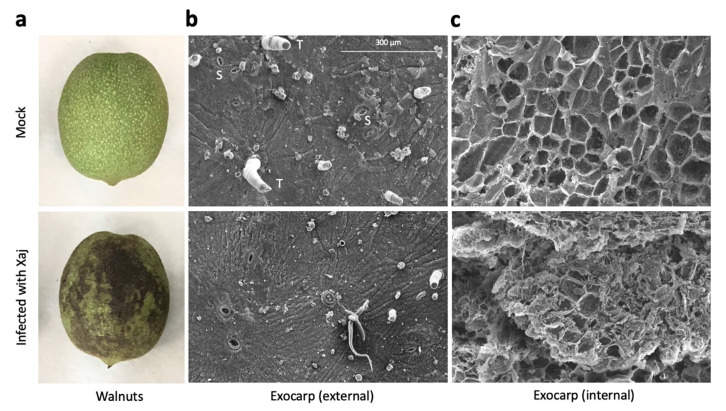
Walnut blight symptoms in walnut fruits. Samples were inoculated with 1 × 10^8^ bacterial cells/mL in a solution of MgCl_2_ and incubated in a box to maintain high humidity at room temperature for ten days under a 12-h light/dark cycle. (**a**) Walnut hull symptom development after seven days. (**b**) Scanning electron micrograph of the external and (**c**) internal-facing exocarp tissues (250×) after ten days. S: stomata and T: trichome.

Controlled Xaj inoculations of walnut fruits closely replicated the typical WB phenotype in the disease symptom development (Figure 1a). The symptom development was quantified and compared to the mock inoculations (Figure S1), followed by microscopy of the exocarp (hull) to determine how the infection affects the surface and cell wall structures of the external-facing and internal layers below the surface of the exocarp (Figure 1b,c). 

**Figure 2:**
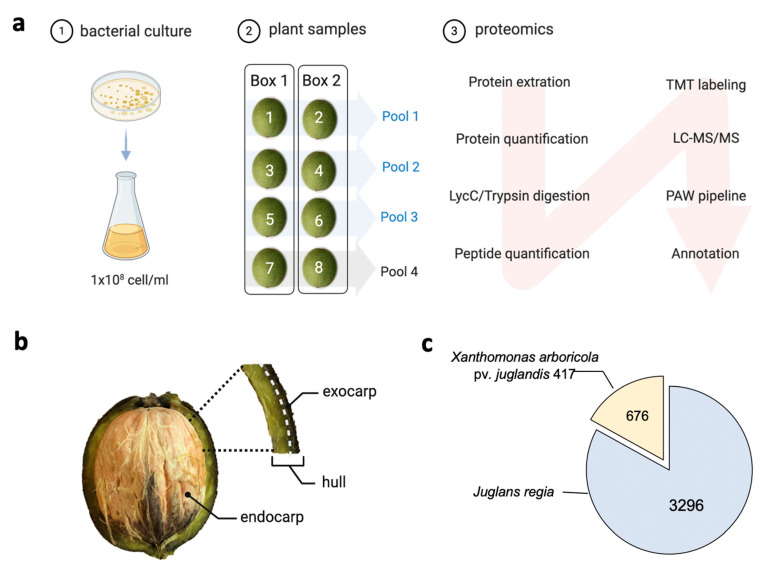
Overview of sample processing. (**a**) Sample preparation and proteomics workflow: (1) inoculations were started by plating Xaj cells in YEP plates and cultivating overnight. Cells were transferred to YEP liquid media and cultured to reach 1 × 10^8^ cells/mL after wash and resuspension in MgCl_2_. (2) The experimental design of nut inoculations and pool formations used for proteomics (Pools 1 to 3) and scanning electron microscopy (Pool 4). (3) The proteomics pipeline used for three pools of each sample type. (**b**) Depiction of the hull material used for microscopy and proteomics. (**c**) Total proteins identified for Jr and Xaj. TMT: tandem mass tag and PAW: proteomic analysis workflow. Created with BioRender.com.

The proteomic analysis identified host and pathogen-derived proteins associated with WB disease in an unbiased manner. The proteome of six Jr hulls infected with Xaj were combined into three pools (two samples per pool) compared to the same number of samples from the mock-inoculated Jr hulls. We cultivated Xaj cells in vitro and inoculated walnut fruits collected from a healthy orchard. Proteins from the infected walnut fruits collected at seven days post-inoculation were compared to mock-inoculated fruits and followed the proteomics workflow shown (Figure 2a). The hull tissue (exocarp) cell morphology was analyzed (Figure 2b). The relative abundance of the detected proteins was compared between the infected and mock samples. After quality-control filtering, 3972 proteins were identified considering a minimum of two mapped peptides (1% peptide spectrum match (PSM) and false discovery rate (FDR)) that contained ten decoy proteins. The final dataset (Figure 2c) comprised 3296 proteins for Jr (Table S1) and 676 proteins for Xaj (Table S2).

### 2.1. Quantitative Proteomic Analysis of Juglans regia in Response to Walnut Blight

**Figure 3:**
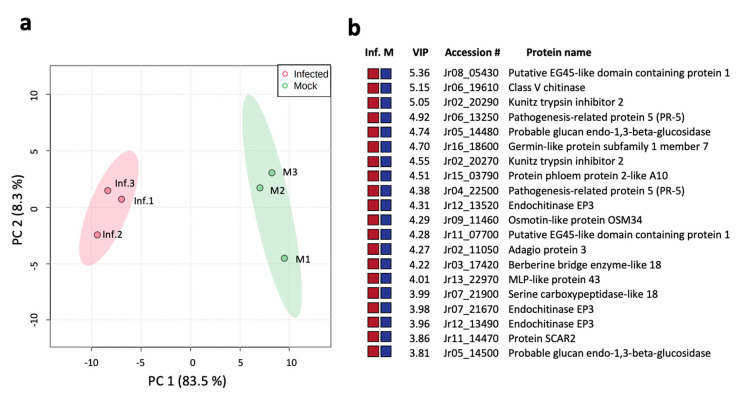
*Juglans regia* proteome analysis comparing the infected and mock-inoculated samples. (**a**) Principle component analysis (PCA) plot with distinct groups and samples. (**b**) Top 20 proteins contributing to the variance between the observed partial least squares-discriminant analysis (PLS-DA) (Figure S2a) with high variable importance in projection (VIP) scores (Table S3), with colored boxes indicating the relative abundance (greater: red and reduced: blue) of the corresponding protein detected in infected (Inf.) and mock-inoculated (M) tissues.

For an unbiased analysis of the proteomic results, the six samples were clustered using principal component analysis (PCA) on the 3296 identified Jr proteins. The two main components explained 91.8% (PC1 83.5% and PC2 8.3%) of the variation between the infected and mock samples, forming two distinct tight clusters according to the sample group (Figure 3a). The heatmap visualization of the proteomic data also showed distinct segregation between the infected and mock-inoculated groups (Figure S2a). The supervised partial least squares-discriminant analysis (PLS-DA) measured the variable importance in projection (VIP) score and estimated the importance of each protein in the PLS model (Figure S2b,c), increasing the relevant biomarker discovery that best explains the difference between the two sample groups. This analysis allowed the identification of the top 20 proteins responsible for group discrimination (Figure 3b). All 20 proteins were more abundant in the infected samples than in the mock-inoculated control (Table S3). They are associated with plant defense, such as putative EG45-like domain-containing proteins (Jr08_05430|109018768 and Jr11_07700|108992001), considered plant natriuretic peptides (PNP); chitinases (Jr06_19610|109012912, Jr07_21670|108980762, and Jr12_13520|108980790); Kunitz trypsin inhibitors (Jr02_20270|109011731 and Jr02_20290|109011765); pathogenesis-related proteins PR2 and PR5 (Jr05_14480|109011271, Jr04_22500|109013108, and Jr06_13250_family|109013532); the germin-like protein subfamily (Jr16_18600|109008986); and MLP-like protein (Jr13_22970|108994024), which were more abundant in the proteome of the infected material (Figure 2b).

**Figure 4:**
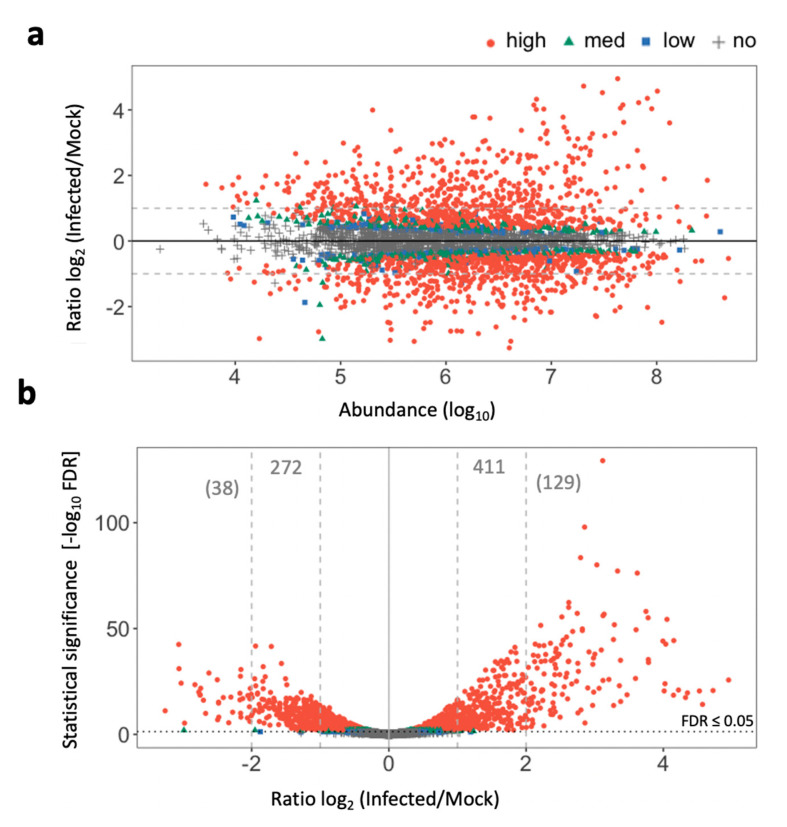
*Juglans regia* proteome in response to Xaj 417 infection showing 3296 detected proteins. (**a**) Ratio-abundance plot. (**b**) Volcano plot analysis of walnut hull infected with Xaj compared to the mock-inoculated hull. Colored dots show false discovery rate (FDR) confidence levels (red for high: FDR ≤ 0.01, green for med: FDR ≤ 0.05, blue for low: FDR ≤ 0.1, and grey for no: FDR > 0.1).

Ratio log_2_ fold-change (log_2_ FC) analysis of the detected proteins in infected and mock samples (Figure 4a) showed 1537 proteins with FDR ≤ 0.01 (high confidence), 322 with FDR ≤ 0.05 (medium), 187 with FDR ≤ 0.1 (low), and 1250 not significantly different (no confidence) than the mock-inoculated control. The protein abundance ranged from 3.29 to 8.68 (log_10_ scale) and log_2_ FC from −3.26 to 4.95 (Figure 4a). The total number of significantly differentially abundant proteins between the two groups was 2046 (FDR ≤ 0.1), of which 1040 were increased (log_2_ FC > 0) and 1006 were decreased (log_2_ FC < 0) in the infected samples. An analysis of statistical significance (volcano plot) of the detected proteins showed 411 (log_2_ FC ≥ 1) increased proteins (right side, Figure 4b), of which 129 were at least four times more abundant (log_2_ FC ≥ 2) than in mock-inoculated nuts, considering a FDR ≤ 0.05. The total of the decreased proteins in the infected tissue was lower, with 272 log_2_ FC ≤ −1, of which only 38 decreased more than four-fold (log_2_ FC ≤ −2) (left side, Figure 4b). 

### 2.2. Quantitative Proteomic Analysis of Xanthomonas arboricola pv. juglandis in Walnut Hulls 

An analysis of WB symptomatic tissue revealed 676 Xaj proteins. Protein abundances from the three replicates representing six nuts were averaged and ranked from the most to the least abundant (Table S2). Due to the number of replicates, the proteins that were not detected in all three pools (117) were removed; some could have biological relevance, such as lipase, chitinase, hemolysin-like, and the copper homeostasis protein CutC. The 20 most abundant differential proteins were determined (Table 1). The most abundant are the OmpA-OmpF, and the list includes well-known PAMPs Ef-Tu and Ax21, GroEL, GroES, glucose dehydrogenase, tetratricopeptide repeat protein, peptidylprolyl isomerase, VirK-like, siderophore, TonB-dependent receptor, glutamine synthetase, ATP synthase, succinyl-CoA synthetase, DNA-binding protein, malate dehydrogenase, peroxiredoxin, cold-shock protein, and a hypothetical protein AKJ12_RS21505 (Table 1).

A functional analysis of the detected proteins shows a high frequency of TonB-dependent outer membrane receptors (32), ATP-binding cassette (ABC) transporters (11), iron uptake and storage systems (7), phosphate uptake systems (3), copper resistance (3), chaperones (5), osmo and redox regulators (17), ribosomal proteins (41), and energy metabolism, such as glycolysis and tricarboxylic acid cycle enzymes (Table S2). Along with the 20 most abundant proteins, it is important to highlight the type III effectors: HopQ (XopQ); XopN; and XopX (AKJ12_RS14220, AKJ12_RS11690, and AKJ12_RS08540) and 67 type II effectors: secreted chorismate mutase (AKJ12_RS15500); three asparaginases (AKJ12_RS06635, AKJ12_RS18355, and AKJ12_RS13110); four peptidyl-prolyl isomerases (AKJ12_RS10980, AKJ12_RS11150, AKJ12_RS06285, and AKJ12_RS11525); beta-glucosidase (AKJ12_RS16605); cell wall degradation proteins such as two polygalacturonases (AKJ12_RS07840 and AKJ12_RS01955), a cellulase (AKJ12_RS14810), two serine proteases (AKJ12_RS11910 and AKJ12_RS21510); proteases/peptidases (33); two esterases (AKJ12_RS20070 and AKJ12_RS13810); endoglucanase (AKJ12_RS08070); two phospholipases (AKJ12_RS07015 and AKJ12_RS03905); five phosphatases (AKJ12_RS09730, AKJ12_RS09895, AKJ12_RS11635, AKJ12_RS05780, and AKJ12_RS13650); alpha-xylosidase (AKJ12_RS09825); xylose isomerase (AKJ12_RS20500); four hydrolases (AKJ12_RS18195, AKJ12_RS14795, AKJ12_RS12205, and AKJ12_RS12930); four aminopeptidases (AKJ12_RS15095, AKJ12_RS13855, AKJ12_RS05625, and AKJ12_RS04085); and biotin carboxylase (AKJ12_RS08585). In addition, we highlighted argininosuccinate synthase (AKJ12_RS01860); lytic murein transglucosylase (AKJ12_RS06545); two copper-binding proteins located adjacent to each other in the Xaj genome; and a copper chaperone (AKJ12_RS00535, AKJ12_RS00530, and AKJ12_RS05060). Some less-described virulence factors that were also detected include VirK-like proteins (AKJ12_RS17315 and AKJ12_RS17320), a single-stranded DNA binding protein (AKJ12_RS12195), a DNA-binding ferritin-like protein (AKJ12_RS16595), an organic hydroperoxide resistance protein (AKJ12_RS18180), and an isochorismatase (AKJ12_RS00070). In addition, 41 hypothetical proteins (among them, 15 with a secretion signal peptide; Table S4) were also identified in the Xaj proteome.

### 2.3. Gene Ontology Annotation and Enrichment Analysis

Among the 411 Jr proteins considered with greater abundance in infected hulls (log_2_ FC > 1 and FDR ≤ 0.05), 329 could be mapped to gene ontology (GO) terms as biological processes, molecular functions, cellular components, protein classes, and pathways using Protein ANalysis THrough Evolutionary Relationships (PANTHER) overrepresentation Fisher’s exact test with FDR correction (Table S5). Based on a fold-enrichment analysis and FDR test (<0.05), we highlighted four of the 21 specific biological processes of particular interest: monosaccharide metabolic process (GO:0005996), alpha-amino acid metabolic process (GO:1901605), small molecule catabolic process (GO:0044282), and organonitrogen compound catabolic process (GO:1901565). The 25 molecular functions overrepresented include copper ion binding (GO:0005507), carbohydrate kinase activity (GO:0019200), lipase activity (GO:0016298), endopeptidase activity (GO:0004175), and oxidoreductase activity (GO:0016491), among other catabolic functions. The main cellular components affected included the cell wall (GO:0005618) and cytosol (GO:0005829). Among the 10 PANTHER protein classes significantly represented are nucleotidyltransferase (PC00174), oxidoreductase (PC00176), protease (PC00190), and the metabolite interconversion enzyme (PC00262). Only two PANTHER pathways were significantly overrepresented: phenylethylamine degradation (P02766) involved in energy metabolism and represented by three amine oxidases and vitamin B6 metabolism (P02787) involved in amino acid interconversion and represented by three pyridoxal reductases.

An analysis of the 272 Jr proteins with reduced abundance in the infected tissues (log_2_ FC < −1 and FDR ≤ 0.05) resulted in 237 mapped proteins (Table S6) overrepresented in biological processes such as chromatin silencing (GO:0006342), ribosomal large subunit biogenesis (GO:0042273), and the monosaccharide metabolic process (GO:0005996), among 61 other classified terms, suggesting an arrest in the biosynthesis and channeling of the carbon and energy sources toward the degradation pathways. Eight molecular functions decreased during infection, including the structural constituents of the ribosome (GO:0003735), RNA binding (GO:0003723), and oxidoreductase activity (GO:0016491). The cellular components most negatively affected among the 29 terms included the ribosomal subunit (GO:0044391), chloroplast (GO:0009507), and cytosol (GO:0005829). The eight PANTHER protein classes highlighted included histone (PC00118), the ribosomal protein (PC00202), and dehydrogenase (PC00092). No specific pathway was statistically overrepresented in the protein set with the reduced abundance in symptomatic tissues.

The gene ontology terms annotated on 540 proteins of 676 in the Xaj proteome detected in planta were mapped, and the functional enrichment analysis highlighted the general trends observed (Table S7). The 56 biological processes included ribonucleoprotein complex assembly (GO:0022618) and the organonitrogen compound biosynthetic process (GO:1901566), among others. Enriched terms related to molecular function included the structural constituent of ribosome (GO:0003735), organic cyclic compound binding (GO:0097159), and oxidoreductase activity (GO:0016491), among 12 terms, and those related to cellular components include 29 significant terms among the ribonucleoprotein complex (GO:1990904) and cell outer membrane (GO:0009279). Eleven PANTHER protein classes were overrepresented and included ribosomal protein (PC00202), peroxidase (PC00180), lyase (PC00144), dehydrogenase (PC00092), and oxidoreductase (PC00176). 

### 2.4. Subcellular Localization

**Figure 5:**
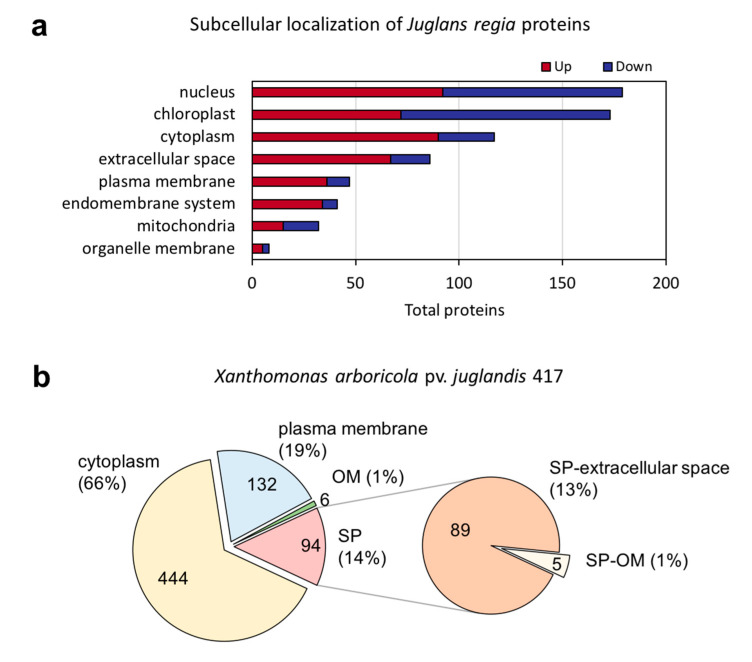
Subcellular localization prediction according to BUSCA. (**a**) Subcellular localization of statistically significant *Juglans regia* proteins with a ratio log_2_ fold-change greater than two (FDR < 0.05). Increased during infection (up) and decreased (down). (**b**) Subcellular localization prediction of the 676 *Xanthomonas arboricola* pv. *juglandis* proteins. OM: outer membrane and SP: signal peptide.

Among the significantly increased (411) and decreased (272) Jr proteins, most were predicted to target the nucleus (179), chloroplast (173), cytoplasm (117), or extracellular space (86), according to the subcellular prediction software BUSCA [41] (Tables S8 and S9). Most nucleus proteins increased (92), while most chloroplast proteins decreased (101) (Figure 5a). Among the Xaj proteins (676), most were located in the cytoplasm (444), the plasma membrane (132), or the outer membrane (6), and 94 had a signal peptide for secretion to the outer membrane (5) or extracellular space (89), according to BUSCA (Figure 5b).

### 2.5. Plant Defense Response to Walnut Bacterial Blight Disease

Ninety-one proteins from the proteome of Jr infected tissue are associated with plant defense (Table 2). Most are members of families with several copies (Table S10) and significantly increased during infection: plant natriuretic peptides (EG45-like domain-containing protein—PNP), Kunitz trypsin inhibitors (KTI), germin-like proteins (GLP), osmotin-like proteins (OLP), patatin-like protein (PLP), and pathogenesis-related proteins (PR1 and PR5). In addition, two proteins essential for ethylene biosynthesis, S-adenosylmethionine synthase (SAM) and 1-aminocyclopropane-1-carboxylate oxidase (ACC), increased significantly during Xaj infection.

There was also increased the abundance of proteins involved in the shikimate pathway and the biosynthesis of phenylpropanoids (Table S11), which synthesized precursors for the production of phytohormone salicylic acid (SA). Thus, we highlighted the significant increase of shikimate dehydrogenase (Jr07_19410), chorismate mutase 1 (Jr12_22090_family), and to phenylalanine ammonia-lyase 2 (Jr12_21910_p1_family). Polyphenol oxidases also increased, particularly JrPPO2 (Jr03_06810), with a log_2_ FC of 3.6 (FDR < 0.01), and JrPPO1 (Jr03_06780), with a log_2_ FC of 0.28 (FDR < 0.05). Twenty-one peroxidases increased with infection (FDR < 0.05). 

## 3. Discussion

Walnut bacterial blight is the most serious above-ground bacterial disease of this tree crop, as the pathogen targets nut tissues, devastating the productivity and quality of walnut orchards. This is the first study that examines the molecular responses during the disease development by comparing the proteomes of infected fruit hulls to healthy tissue. This study was leveraged by the complete genome sequences from the pathogen and the recently published chromosome-scale reference walnut genome [40] to more accurately predict the contribution of the specific plant or pathogen-derived proteins and determine their role in WB disease. The degradation of plant tissues in infected fruits, shown by the scanning electron microscopic analysis, can now be interpreted from a proteomic perspective, as formed by the plant-pathogen interaction. Describing the contributions and interplay of thousands of individual proteins is no trivial task; however, the proteome data identifies major contributors that deserve further discussion and characterization.

From the plant host perspective, chitinases and GLPs were among the top 20 proteins identified by PLS-DA as the best at differentiating infected from mock-inoculated samples. An increased abundance of germin-like proteins (GLPs) in WB could be contributing to increased basal defense mechanisms, which are conserved in many plants and involve enzymatic activities like superoxide dismutase (SOD), oxalate oxidase, and polyphenol oxidase (PPO) [42]. Our results show that the host responses detected in WB are conserved in other *Xanthomonas* pathosystems, in which chitinases and GLPs in tolerant rice seedlings are prominent features. They potentially increase the plant resistance or tolerance to stress, as in the proteome of rice leaf blight, caused by *Xanthomonas oryzae* pv. *oryzae* (Xoo) [43]. The presence of high levels of PPO activity in the hulls of Jr is consistent with its role in producing reactive quinones [44] and regulating the production of phenylpropanoid biosynthesis and cell death control [38]. Based on our proteomic data, we previously showed that Jr PPOs were differentially expressed during infection, contributing to its role in the defense response [34]. 

It is important to mention the reduced abundance of translational machinery during infection, which may be a defense mechanism against infection [45]. The large increase in plant natriuretic peptides (PNPs) during WB is potentially linked to their role in plant homeostasis, where they work as signal molecules secreted in the apoplast during infection. PNP-dependent responses include tissue-specific modifications dependent on cation transport, changes in photosynthesis and respiration rates, and stomatal conductance during stress [46,47]. *Xanthomonas axonopodis* pv. *citri* (Xac) uses a PNP-like protein to modify host homeostasis [48]. Xac PNP shares significant sequence similarity and the same domain composition with plant PNPs but has no homologs in other bacteria. However, Xaj 417 does not have the PNP homolog from Xac or any protein similar to the three PNPs identified in the Jr proteome.

**Figure 6:**
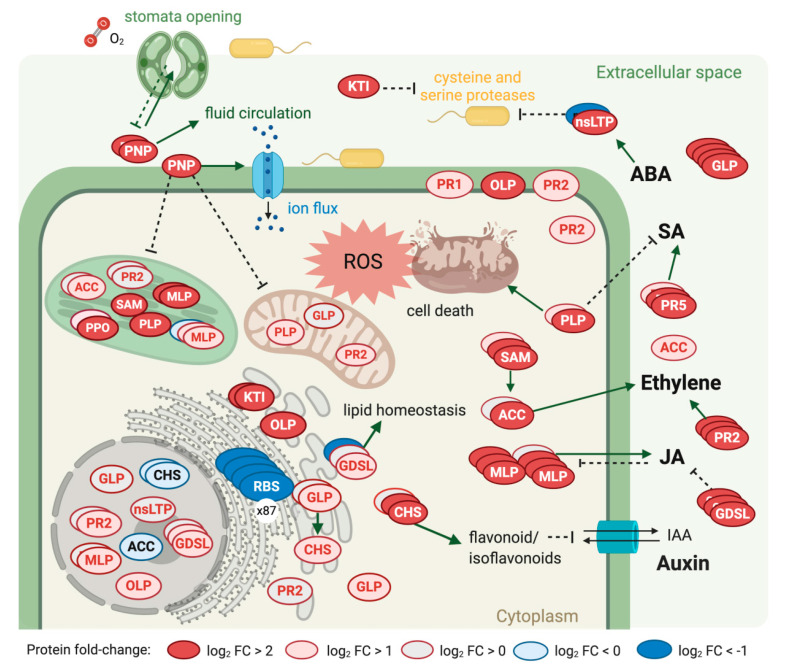
Walnut bacterial blight plant response following Xaj 417 infection. Copies of proteins from the same family detected in proteomics are represented. ACC: 1-aminocyclopropane-1-carboxylate oxidase, CHS: chalcone synthase, GDSL: GDSL lipase/esterases, GLP: germin-like proteins, KTI: Kunitz trypsin inhibitor, MLP: major latex proteins, nsLTP: plant nonspecific lipid-transfer protein, OLP: osmotin-like protein (PR5-like), PLP: patatin-like protein, PNP: plant natriuretic peptides, PR1: pathogenesis-related 1, PR2: glucan endo-1,3-beta-glucosidase, PR5: pathogenesis-related 5, SAM: S-adenosylmethionine synthase, JA: jasmonic acid, SA: salicylic acid, and ROS: reactive oxygen species. Created with BioRender.com.

Among the increased proteins from the host, we highlighted the defense proteins associated with phytohormone biosynthesis, including salicylic acid (SA), jasmonic acid (JA), and ethylene. These proteins are associated with either the induction or repression of these hormones [49,50,51]. SA is involved in basal resistance or systemic acquired resistance (SAR) by inducing the expression of pathogenesis-related (PR) genes [52]. The proteome of infected Jr showed a significant increase in important SAR induction markers, including the PR1, PR2, PR3, and PR5 proteins, mostly predicted to be located in the extracellular space. The biosynthesis of SA depends on the conversion of phenylalanine to trans cinnamic acid by phenylalanine ammonia-lyase 2 (PAL). This is a key enzyme of plant metabolism, catalyzing the first reaction in biosynthesis from L-phenylalanine of a wide variety of natural products based on the phenylpropane skeleton. The GO analysis showed enrichment of the phenylethylamine degradation pathway. The abundance of many proteins involved in phenylpropanoid biosynthesis increased during infection to boost the immune response and fight the bacterial infection. However, due to the observed symptoms, this increase was insufficient to combat the Xaj virulence factors. Intriguingly, a gene located in a genomic island encodes an isochorismatase (AKJ12_00070) that may suppress salicylate-mediated innate immunity in plants by the diverting of the plant salicylate metabolism and converting of SA into catechol. The borders of this genomic island (comprising AKJ12_RS00015-AKJ12_RS00145) is flanked by an integrase and a transposase, indicating its acquisition through horizontal gene transfer. An isochorismatase secreted by *Phytophthora sojae* and *Verticillium dahliae* is required for full pathogenesis [53]. Moreover, they lack known signal peptides, although they show characteristics that lead to an unconventional secretion that could be a novel mechanism for delivering effectors and may play an important role in host-pathogen interactions [54]. Zhu and collaborators showed that isochorismatase from *V. dahliae* is a virulence factor that interferes with salicylate and jasmonate defense signaling [54]. A depiction of the interplay of all these molecular responses on the host side is presented (Figure 6).

**Figure 7:**
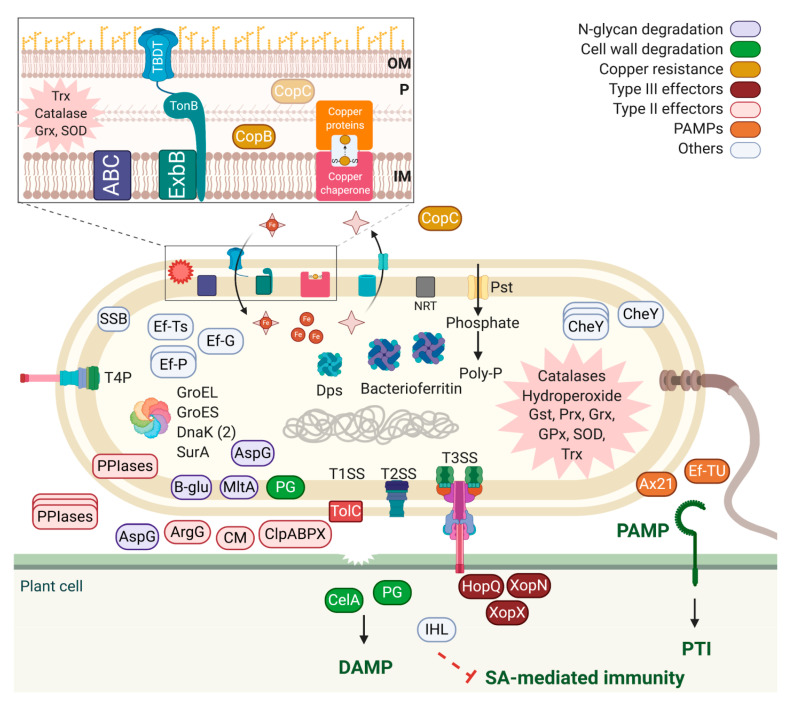
Role of Xaj 417 proteins in disease development. OM: outer membrane, IM: inner membrane, P: periplasm, SSB: single-stranded DNA binding, PPIases: peptidyl-prolyl isomerases, AspG: asparaginase, B-glu: beta-glucosidase, MltA: murein-degrading enzyme, PG: polygalacturonase, ArgG: argininosuccinate synthase, CM: secreted chorismate mutase, Clp: proteases, CelA: cellulase, IHL: isochorismatase-like hydrolases, CheY: chemotaxis, NTR: nitrate transporter, Dps: DNA-binding ferritin-like, Trx: thioredoxin, Grx: glutaredoxin, SOD: superoxide dismutase, Gst: glutathione transferase, Prx: peroxiredoxin, and GPx: glutathione peroxidase. Created with BioRender.com.

Six hundred and seventy-six proteins, which comprise ~16.2% of the Xaj genome, were identified in the infected walnut proteome. The most abundant was OmpA-OmpF, a porin associated with evasion of the host defense [55]. It is well-conserved among Xanthomonadaceae species, and its sequence is 70% identical to *Xylella fastiodiosa*’s MopB sequence, a protein associated with sensitivity to oxidative stress [56,57] and required for the pathogenicity of *Xanthomonas campestris* pv. *campestris*, the causal agent of black rot in crucifers [58]. It was possible to identify Xaj proteins related to evasion of the host defense, protection against oxidative stress, adaptation and virulence, and PAMPs such as Ef-Tu and Ax21: extracellular proteins capable of triggering host immune responses during an infection. These PAMPs are conserved in nonpathogenic Xaj strains [59]. The Xaj 417 molecular strategies detected in infected walnuts leading to WB are summarized (Figure 7). Extracellular proteins such as Ef-Tu and Ax21 were among the most abundant proteins, along with others involved with chemotaxis (CheY) and type IV pilus function (PilT, PilQ, PilB, PilG, PilM, and PilZ). A cellulase, a murein-degrading enzyme, two polygalacturonases, and proteases/peptidases are also abundant in infected tissue, with 39 peptidases and proteases being the most prominent.

**Figure 8:**
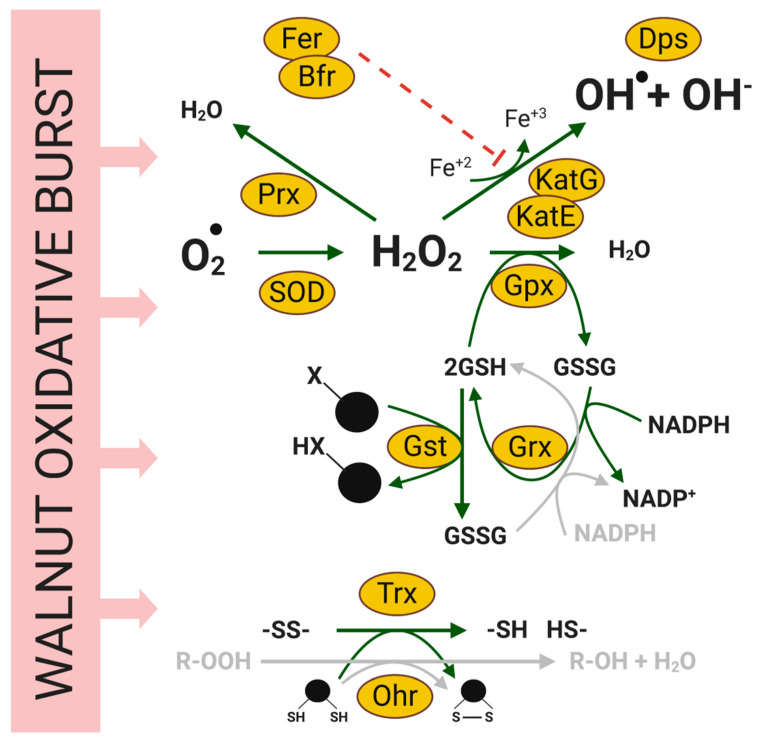
Oxidative stress in response to walnut bacterial blight. Reactive oxygen species (ROS) enzymes represent bacterial proteins identified in the Xaj proteome. Fer: ferritin, Bfr: bacterioferritin, Dps: DNA-binding proteins/ferretins, Ohr: organic hydroperoxide resistance protein, Trx: thioredoxin, Kat: catalase, SOD: superoxide dismutase, Prx: peroxiredoxin, Gpx: glutathione peroxidase, Grx: glutaredoxin, Gst: glutathione transferase.

Important components of the interaction network leading to WB include type II secreted proteins found in the Xaj proteome: polygalacturonases, cellulase, serine proteases, proteases/peptidases, esterases, endoglucanase, phospholipases, phosphatases, alpha-xylosidase, xylose isomerase, hydrolases, aminopeptidases, and biotin carboxylase [60,61]. The type II secreted proteins are well-characterized in *Xanthomonas* spp. [62,63,64], and many of these proteins are cell wall-degrading enzymes that contribute to bacterial virulence, translocate virulence factors into the plant cells, and facilitate the acquisition of nutrients [65,66]. In addition to type II effectors, Xaj also employs type III effectors, some of which were detected in infected tissues. These include HopQ (XopQ), XopN, and XopX, which suppress the host immune responses [67,68]. In Xoo, XopN is a virulence factor that interacts with two rice proteins, a zinc finger protein 2 (OsVOZ2), and a XopNKXO85 binding protein (OsXNP) [69], which also decreases in the Jr proteome as thiamine thiazole synthase (Jr09_15000|108993518). In addition, XopN may suppress PTI in tomatoes [70]. Here, we confirmed that these three type III effectors, which are absent in nonpathogenic *X. arboricola* pv. *juglandis* strains (unpublished), are expressed in tissues developing WB. An analysis of Xaj proteins associated with metabolic activities revealed that glycolysis and the tricarboxylic acid cycle are fully activated. Likewise, it was possible to identify at least 17 proteins associated with adaptation to oxidative stress, which corroborates the plant’s ability to produce reactive oxygen species as a primary plant defense mechanism (Figure 8). In addition, the identification of proteins associated with different secretory systems, the type IV pilus structure, and the ability to internalize metabolites and ions from plant tissue denotes Xaj’s ability to adapt to the adverse conditions imposed by the host, which characterizes Xaj as a well-adapted and versatile pathogen.

Xaj 417 is a copper-resistant strain isolated from a diseased tree in California [39], where the intensive use of copper sprays over a century led to the evolution of copper-resistant strains. The presence of copper resistance proteins from the same genomic region indicates that this bacterium may have acquired this cluster through horizontal gene transfer, as suggested for other copper- resistant *Xanthomonas* species [59,71,72,73]. Among the copper-binding proteins, a copper chaperone was also identified in this study. This class of proteins is responsible for delivering copper ions to copper-binding proteins [74]. The copper ion-binding function was also enriched in the Jr proteome of the infected tissue. Among the adaptive mechanisms and virulence induction, lipopolysaccharide (LPS) modulation stands out as a mechanism to evade host recognition and activation of the plant defense, in addition to the previously mentioned type II and III effectors. None of the 21 Jr SWEET transporters identified by [34] were identified in this study, which corroborates the lack of TALEs in Xaj 417 and lack of activation of Jr SWEET under infection with Xaj 417 [34].

Still, in the theme of host immune evasion, our results confirmed that Xaj employs a secreted chorismate mutase as an important component of its pathobiology. A computational analysis found that the encoding gene is a characteristic of plant-associated Xanthomonadaceae (*Xanthomonas* and *Xylella* spp.) [63], and we now demonstrated its expression in planta. Chorismate mutase is a key enzyme in the shikimate pathway responsible for aromatic amino acid synthesis. Several pathogenic bacteria code for a secreted chorismate mutase, a type II effector that may be associated with a decreasing SA production in the host by displacing the pathway to aromatic amino acids [63,75]. Although the role of this secreted chorismate mutase in virulence is unknown, Xoo knockout mutants are hypervirulent to rice [76]. Another interesting protein is argininosuccinate synthase (ArgG). Arginine is an essential amino acid for bacterial metabolism and can be either provided by the host or synthesized by bacterial cells. The inactivation of ArgG in Xoo affected the bacterial growth, and symptoms in leaves of rice inoculated with the mutant were less symptomatic [77]. In the secretome study of a hypervirulent mutant strain of *Xylella fastidiosa* lacking the protease PrtA, the most upregulated proteins were ArgG and ArgH [78]. These proteins are involved in arginine biosynthesis and nitrogen metabolism. 

Finally, VirK-like proteins (another characteristic of plant-associated Xanthomonadaceae) were highly abundant in diseased tissues, but their precise role remains elusive. Another protein worth noting is AKJ12_RS13340. Although of unknown function, this protein is a member of a functionally diverse superfamily containing proteases, lipases, peroxidases, esterases, epoxide hydrolases, and dehalogenases. This specific protein has only been found in members of *X. arboricola*. Another uncharacterized protein that deserves further investigation is AKJ12_RS15060, a YadA domain-containing protein, and a member of the homotrimeric adhesin XadA family [79]. As reported, most proteins identified in the Jr proteome during infection reveal their responses as a result of the attack induced by Xaj. This pathogen proves highly versatile by activating a repertoire of proteins that induce damage to the plant structure at the same time that it defends itself from the chemical attacks of its host. This is the first time that the molecular interaction in this pathosystem was revealed, with potential pathways and target proteins for the improvement of *J. regia* resistance to WB and identifying new Xaj proteins that could be targeted for rational drug development, thereby decreasing cupric management.

## 4. Materials and Methods 

### 4.1. Plant Material and Nut Inoculations with Xanthomonas Arboricola pv. Juglandis 417

Eight nuts from *Juglans regia* cultivar Chandler were collected randomly from Hutchinson Field at the University of California, Davis, in July 2019. Nuts were washed with distilled (DI) water to remove dust and dried with clean tissue paper. Bacterial culture of Xaj 417 was grown on a YEP plate for two days previous to the inoculation. A small chunk of cells was transferred to a 50-mL Falcon tube with 20-mL YEP (two replicates) and grown overnight at 27 °C in a shaker at 200 rpm. Cultures were centrifuged for 10 min at 4000× *g* to remove culture medium. The supernatant was discarded, and the pellet was washed with 15-mL inoculation solution (5-mM MgCl2 with 0.1% BREAK-THRU surfactant). The OD600 was measured and cultures were centrifuged under the same conditions, and the pellet was washed with 10 mL of the same solution. After the second wash, the volumes from the two replicates were mixed to increase the number of cells. OD600 was adjusted to 10^8^ cells/mL in 245-mL inoculation solution. For inoculation, the nuts were submitted to vacuum in the bacterial solution for 1 min (30 s of vacuum on and 30 s under pressure), positioned to dry on a tissue paper, then placed over a grid in two humidity boxes under a 12-h light cycle. The development of symptoms was tracked over ten days, but the material was collected at the seventh day for proteomic and at the tenth for microscopic analyses. Symptoms were measured by quantifying the dark spot area on the surface of each fruit using ImageJ 1.50i [80].

### 4.2. Scanning Electron Microscopy

Walnut hull (exocarp) discs were fixed overnight in 0.1-M sodium phosphate buffer with 2.5% glutaraldehyde and 2% paraformaldehyde. Tissues were rinsed three times in 0.1M NaH2PO4 for 10 min and dehydrated in increasing concentrations of ethanol (EtOH) for 10 min each (30, 50, 70, 95, and 100% EtOH). Dehydration with 95% and 100% EtOH was repeated three times. Samples were dried using Tousimis 931 GL Super Critical Autosamdri, mounted onto aluminum stubs and coated with gold using Pelco Auto Sputter Coater SC-7. Micrographs were produced on a Thermo Fisher Quattro S Environmental operating at 15 kV at the UC Davis Advanced Materials Characterization and Testing laboratory (AMCaT).

### 4.3. Protein Extraction and Quantification

Protein extraction was performed based on a previously described phenol extraction protocol [81] with modifications. The exocarp was removed from the whole fruit. Pools were made from the material of two fruits (one from each box), frozen immediately with liquid nitrogen, and stored at −80 °C. Frozen plant tissue was ground to a fine powder with a mortar and pestle in liquid nitrogen. About one gram of the fine frozen powder was suspended in 3-mL extraction buffer (500-mM Tris-HCl, 50-mM EDTA, 700-mM sucrose, 100-mM KCl, 2% β-mercaptoethanol, 1-mM PMSF, and SigmaFAST™ protease inhibitor cocktail). The suspension was homogenized and incubated on ice under shaking at 100 rpm for 10 min. An equal volume of Tris-buffered phenol, pH 7.0, was added and homogenized on a shaker at 100 rpm at room temperature for 10 min. The samples were centrifuged at 5500× *g* for 10 min at 4 °C, and the upper phenol phase was carefully recovered and poured into a new tube. This phenol phase was used for re-extraction in 3 mL extraction buffer, followed by homogenization for 3 min, vortexing, and centrifugation at the same configuration used previously for phase separation. The new phenol phase was carefully recovered and poured into a new tube. Proteins were precipitated using four volumes of cooled precipitation solution (100-mM ammonium acetate in methanol) overnight at −20 °C, followed by centrifugation at 5500× *g* for 10 min at 4 °C. Protein pellets were washed three times with 1-mL cooled precipitation solution and one time with 300-µL cooled acetone. The pellet was dried 1 h in the hood and 1 min under vacuum. Proteins were solubilized with 150-µL urea buffer (6-M urea and 50-mM triethylammonium bicarbonate). For protein quantification, 10 µL was used for a 1:10 dilution to determine the protein concentration using the Pierce™ BCA Protein Assay Kit (Thermo Fisher Scientific, Waltham, MA, USA) using bovine serum albumin (BSA) as a standard.

### 4.4. Protein Digestion, Tandem Mass Tag Labeling, and Mass-Spectrometry

The following steps were carried out with 100-µg crude protein extract from each sample. Samples were reduced with dithiothreitol (DTT) to a final concentration of 5 mM and incubated at 37 °C for 30 min at 1000 rpm. Alkalization was done with iodoacetamide (IAA) to a final concentration of 15 mM, and the samples were incubated for 30 min at 1000 rpm at room temperature in the dark. The quenching step was done using 20 µL of 200-mM DTT incubated at room temperature for 10 min. Three µg of Lys-C protease was added to each sample, and they were incubated at 30 °C for two hours at 1000 rpm, followed by dilution with 550 µL of 50-mM triethylammonium bicarbonate (TEAB). Digestion was done with 2-µg trypsin at 1000 rpm at 37 °C for 2 h, followed by the addition of 2-µg trypsin, and continued the incubation overnight at 37 °C and 1000 rpm. The sample volume was reduced to 50 µL using a speed vacuum concentrator for three hours. Samples were acidified with 10% trifluoroacetic acid (TFA) and cleaned up with a Macrospin Column C18.

The tryptic extracts from different samples were labeled using the tandem mass tag (TMT) system TMT10plex™ Label Reagent Set (Thermo Fisher Scientific, Waltham, MA, USA) and resuspended according to the manufacturer’s instructions. Two reference pools were made using equal volumes of each sample for the LC-MS/MS. All samples were fractionated using the Pierce™ High pH Reversed-Phase Peptide Fractionation Kit (Thermo Fisher Scientific, Waltham, MA, USA) and submitted to the UC Davis Proteomics Core Facility for LC-MS/MS analysis.

### 4.5. Proteomic Analysis Workflow Pipeline and Statistical Analysis

This experiment was part of a broad study of 16 samples that included nuts inoculated with wild-type Xaj 417 and nuts inoculated with three other mutant bacterial strains that will be discussed in later publications. Mock-inoculated and non-inoculated nuts were used as negative controls of the plant response to the pathogen. The Thermo binary instrument files were processed with MS Convert from the Proteowizard toolkit [82] and Python scripts from the PAW (proteomic analysis workflow) pipeline [83] to create MS2 format files [84] for database searching and to extract the reporter ion peak heights from individual MS3 scans. Database searching used Comet (version 2016.01 rev. 3) [85] with the PAW pipeline for peptide spectrum match (PSM) validation using the target/decoy method [86]. Search parameters included: parent ion monoisotopic mass tolerance 1.25 Da, fragment ion monoisotopic mass tolerance 1.0005 Da, trypsin cleavage with up to two missed cleavages, variable oxidation of Met residues, fixed alkylation of Cys residues, and fixed TMT reagent masses (+229.1620 Da) at peptide N-terminus and Lys residues. The protein database was a combination of *Juglans regia* (41,103 sequences downloaded from NCBI assembly GCF_001411555.2) and *Xanthomonas arboricola* pv. *juglandis* 417 (4178 sequences downloaded from NCBI RefSeq NZ_CP012251.1). Common contaminants (179 sequences) and sequence-reversed decoys were also appended. PSMs were filtered to a 1% false discovery rate (FDR) and mapped to proteins using basic parsimony rules. Protein identifications required at least two distinct PSMs per protein per TMT plex. An additional extended parsimony protein grouping step was used to combine highly homologous proteins into the final list of identified proteins. Shared or unique peptide status was defined in the context of the final protein list. Reporter ion intensities from PSMs associated with unique peptides were summed into protein total intensity values. Protein intensities within each TMT plex and between plexes were normalized using the internal reference scaling (IRS) method [87]. Differential expression testing was done in Jupyter notebooks (https://jupyter.org/) using an R kernel and the Bioconductor package edgeR [88]. Data after IRS was processed by using the trimmed mean of M-values compositional normalization [88], experiment-wide trended variance, pairwise exact test, and Benjamini-Hochberg multiple testing corrections. The normalized abundance data was uploaded to MetaboAnalyst 4.0 (http://www.metaboanalyst.ca) for principal component analysis (PCA), heatmap, and partial least squares-discriminant analysis (PLS-DA) to identify proteins contributing to the discrimination between different treatments. The variables were mean-centered and divided by the standard deviation of each for data scaling; then, the leave-one-out cross-validation (LOOCV) method was performed to evaluate the quality of the resulting statistical models by considering the diagnostic measures R^2^ and Q^2^.

### 4.6. Protein Functional Analyses

Gene ontology (GO) analysis was performed with the PANTHER classification system version 14 [89], using *Xanthomonas campestris* pv. *campestris* strain ATCC 33913 (taxid:190485) orthologs for Xaj proteins and *Arabidopsis thaliana* orthologs for Jr proteins. Fisher’s exact test was performed to assign statistical overrepresentation using FDR and *p*-values <0.05. GO terms were mapped for the functional enrichment analysis using PANTHER.

Annotation information for the identified walnut proteins was added from *Juglans regia* (Chandler v2.0, [40], shorturl.at/mqBM1) and *Arabidopsis thaliana* orthologs (UniProt Swiss-Prot records, 15,814 sequences, 2018.10 release) determined using PAW_BLAST scripts (https://github.com/pwilmart/PAW_BLAST) and an annotation script (https://github.com/pwilmart/annotations). The *Xanthomonas arboricola* pv. *juglandis* strain 417 (Xaj 417) genome sequence and annotation data are deposited at DDBJ/EMBL/GenBank under the accession number CP012251 [39].

The subcellular localization for Jr proteins was predicted by BUSCA [41] and, for Xaj proteins, also SignalP [90] and Phobius [91]. Metabolic pathways were based on KEGG pathways information [92].

## 5. Conclusions

A thorough understanding of the mechanisms driving the evolution of plant-pathogen interactions is essential to understand bacterial susceptibility and plant resistance. The first proteome study of Jr infected with Xaj, a high genetic diversity group among *Xanthomonas*, provided insights into the disease development in this pathosystem. Plant natriuretic peptides (PNPs) were abundant in the walnut hulls during Xaj infection. Many defense proteins increased, including pathogenesis-related and oxidative stress response proteins and those critical for the biosynthesis of key phytohormones (SA, JA, ET, and ABA) against stresses. On the other hand, Xaj proteins detected in infected tissues demonstrated the remarkable ability of this bacterium to adapt to the host microenvironment, adjusting to a limited iron availability and redox conditions, coping with copper toxicity (sprayed to reduce walnut blight), and maintaining the energy and intermediary metabolism and ribosomal function. The secreted proteases and other extracellular appendages such as type IV pilus for twitching motility, type III secretion machinery, and some of its effectors also gave hints of putative factors recognized by the plant host. The results reinforced the importance of type II secreted proteins in disease development, in contrast to other *Xanthomonas* pathogens well-known for manipulating host responses by secreting TALE effectors.

## Figures and Tables

**Table 1:** Top 20 most abundant Xaj 417 proteins in infected walnut hulls. SP: signal peptide.

GenBank ID	Locus Xaj417	Description	SignalP	BUSCA Prediction	Rank Abundance
AKU49410.1	AKJ12_RS06245	OOP family OmpA-OmpF porin	OTHER	Cytoplasm	1
AKU49462.1	AKJ12_RS14455	Elongation factor Tu	OTHER	Cytoplasm	2
AKU49820.1	AKJ12_RS08545	Molecular chaperone GroEL	OTHER	Cytoplasm	3
AKU51588.1	AKJ12_RS18640	Ax21 family protein	OTHER	Cytoplasm	4
AKU50680.1	AKJ12_RS13495	Siderophore	SP(Sec/SPI)	SP-Extracellular space	5
AKU49415.1	AKJ12_RS06280	Malate dehydrogenase	OTHER	Cytoplasm	6
AKU51241.1	AKJ12_RS16600	Glucose dehydrogenase	OTHER	Cytoplasm	7
AKU48616.1	AKJ12_RS01595	30S ribosomal protein S1	OTHER	Cytoplasm	8
AKU51756.1	AKJ12_RS19625	Tetratricopeptide repeat protein	OTHER	Cytoplasm	9
AKU51362.1	AKJ12_RS17320	VirK-like protein	OTHER	Cytoplasm	10
AKU50394.1	AKJ12_RS11890	TonB-dependent receptor	SP(Sec/SPI)	SP-Extracellular space	11
AKU52070.1	AKJ12_RS21505	Hypothetical protein	OTHER	OM-Beta Strand	12
AKU51605.1	AKJ12_RS18740	Glutamine synthetase	OTHER	PM-Alpha Helix	13
AKU49821.1	AKJ12_RS08550	Molecular chaperone GroES	OTHER	Cytoplasm	14
AKU50456.1	AKJ12_RS12245	Peroxiredoxin	OTHER	PM-Alpha Helix	15
AKU51052.1	AKJ12_RS15520	ATP F0F1 synthase subunit alpha	OTHER	PM-Alpha Helix	16
AKU50723.1	AKJ12_RS16695	Succinyl-CoA synthetase subunit alpha	OTHER	Cytoplasm	17
AKU49376.1	AKJ12_RS06020	DNA-binding protein HU	OTHER	Cytoplasm	18
AKU49010.1	AKJ12_RS03895	Cold-shock protein	SP(Sec/SPI)	SP-Extracellular space	19
AKU50240.1	AKJ12_RS10980	Peptidylprolyl isomerase	OTHER	Cytoplasm	20

**Table 2:** *J. regia* proteins associated with the defense response (complete table in the Supplemental Materials, Table S10).

Protein Family	Code	Copies	Ave. Ratio log_2_ (Inf./Mock)	Direction	Ave. FDR
EG45-like domain-containing protein	PNP	3	4.07	up	1.05 × 10^−26^
Kunitz trypsin inhibitor	KTI	3	4.05	up	2.30 × 10^−15^
Pathogenesis-related 5	PR5	3	3.46	up	9.96 × 10^−22^
Osmotin-like protein	OLP	3	2.89	up	9.72 × 10^−19^
S-adenosylmethionine synthase	SAM	4	2.04	up	2.71 × 10^−13^
Major latex like-protein	MLP	15	1.87	up	3.92 × 10^−3^
Patatin-like protein	PLP	4	1.84	up	1.08 × 10^−4^
1,3-beta-glucosidase	PR2	11	1.71	up	6.14 × 10^−2^
Germin-like protein	GLP	10	1.69	up	8.26 × 10^−3^
GDSL esterase/lipase	GDSL	14	1.14	up	1.23 × 10^−1^
Pathogenesis-related 1	PR1	1	1.14	up	3.67 × 10^−7^
Chalcone synthase	CHS	5	0.99	up	5.96 × 10^−3^
1-aminocyclopropane-1-carboxylate oxidase	ACC	9	0.69	up	9.48 × 10^−2^
Non-specific lipid-transfer protein	nsLPT	6	−0.46	down	2.09 × 10^−2^

## Supplementary Materials

The supplementary materials can be found at https://www.mdpi.com/1422-0067/21/20/7453/s1. Figure S1. Walnut blight symptoms in fruits. Figure S2. Jr protein abundance of sample groups. Table S1. Complete *Juglans regia* proteome of diseased and mock samples and annotations. Table S2. Proteome of 676 proteins of *Xanthomonas arboricola* pv. *juglandis* 417. Table S3. Complete list of PLS-DA - VIP scores for *Juglans regia*. Table S4. Hypothetical proteins from Xaj identified in infected walnuts. Table S5. PANTHER Overrepresentation Test of 411 proteins with increased abundance. Gene ontology analysis of Biological Process, Molecular Function, Cellular Component, PANTHER Class, and PANTHER pathway. Table S6. PANTHER Overrepresentation Test of 272 proteins with reduced abundance. Gene ontology analysis of Biological Process, Molecular Function, Cellular Component, PANTHER Class, and PANTHER pathway. Table S7. PANTHER Overrepresentation Test of 676 proteins of Xaj with increased abundance. Gene ontology analysis of Biological Process, Molecular Function, Cellular Component, PANTHER Class, and PANTHER pathway. Table S8. 411 proteins with log_2_ FC ≥ 1 and FDR < 0.05 (high and med) comparing walnuts inoculated with Xaj with mock. Values shown are normalized protein abundance. Table S9. 272 proteins with log_2_ FC ≤ −1 and FDR < 0.05 (high and med) comparing walnuts inoculated with Xaj with mock. Table S10. *Juglans regia* proteins associated with defense response. Table S11: Shikimate and phenylpropanoid biosynthesis proteins.
